# *Frankliniella occidentalis* facilitate *Salmonella enterica* survival in the phyllosphere

**DOI:** 10.1371/journal.pone.0247325

**Published:** 2021-02-19

**Authors:** Victoria L. Harrod, Russell L. Groves, Matthew A. Maurice, Jeri D. Barak

**Affiliations:** 1 Department of Entomology, University of Wisconsin - Madison, Madison, Wisconsin, United States of America; 2 Department of Plant Pathology, University of Wisconsin - Madison, Madison, Wisconsin, United States of America; Banaras Hindu University, INDIA

## Abstract

The human enteric bacterial pathogen *Salmonella enterica* causes approximately 1.35 million cases of food borne illnesses annually in the United States. Of these salmonellosis cases, almost half are derived from the consumption of fresh, raw produce. Although epiphytic *S*. *enterica* populations naturally decline in the phyllosphere, a subset of phytophagous insects have recently been identified as biological multipliers, consequently facilitating the growth of bacterial populations. We investigated whether tomato leaves with macroscopic feeding damage, caused by infestation of adult Western flower thrips (*Frankliniella occidentalis*), support higher *S*. *enterica* populations. To explore this hypothesis, we assessed *S*. *enterica* populations in response to thrips feeding by varying insect density, plant age, and the gender of the insect. As a reference control, direct leaf damage analogous to thrips feeding was also evaluated using directed, hydraulic pressure. In a supplementary set series of experiments, groups of *F*. *occidentalis* infested tomato plants were later inoculated with *S*. *enterica* to determine how prior insect infestation might influence bacterial survival and persistence. Following an infestation period, leaves visibly damaged by adult *F*. *occidentalis* supported significantly higher *S*. *enterica* populations and resulted in greater amounts of electrolyte leakage (measured as electrical conductivity) than leaves lacking visible feeding damage. Plant age did not significantly influence *S*. *enterica* populations or estimates of electrolyte leakage, independent of initial infestation. Additionally, the gender of the insect did not uniquely influence *S*. *enterica* population dynamics. Finally, applications of aggressive water bombardment resulted in more electrolyte leakage than leaves damaged by *F*. *occidentalis*, yet supported comparable *S*. *enterica* populations. Together, this study indicates that *F*. *occidentalis* feeding is one of the many potential biological mechanisms creating a more habitable environment for *S*. *enterica*.

## Introduction

In the United States alone, the human enteric bacterial pathogen *Salmonella enterica* is estimated to cause 1.35 million cases of food-borne illnesses annually (Centers for Disease Control and Prevention, 2020). While it is generally perceived that cases of salmonellosis are acquired from consumption of *S*. *enterica-*contaminated animal products, cross-contamination of fresh produce has become an overwhelming risk throughout the cultivation process, subsequently resulting in a growing rate of produce-associated salmonellosis over the past decade [1, 2]. According to the CDC, *S*. *enterica* is responsible for the majority of bacterial foodborne illness and, unlike other bacterial foodborne pathogens, the incidence of outbreaks has not diminished over the last decade. The switch in vehicles from contaminated animal products to fresh produce is posited as the leading reason for the ongoing salmonellosis outbreak occurrence. Identifying the biotic mechanisms that allow enteric human pathogens, such as *S*. *enterica*, to persist or grow on fresh produce pre-harvest is the first step in reducing the disease burden and increasing food safety.

Harsh environmental conditions, such as direct UV radiation or lack of available nutrients, make the phyllosphere of plants an adverse environment for *S*. *enterica* [3]. In fact, *S*. *enterica* populations have been observed to naturally decline on healthy leaves [4–6]. In spite of these challenges, *S*. *enterica* populations must persist or find advantageous niches or partnerships on plants in the field pre-harvest, because salmonellosis outbreaks from consumption of raw produce continue to occur regularly. In addition to agricultural practices acting as bacterial reservoirs [7], insects are also recognized as potential biological vectors of *S*. *enterica* given their unique behaviors as they facilitate the dispersal and enhance the persistence of the bacterium [8]. Multiple species of cockroaches and darkling beetles, for instance, phoretically transfer or mechanically vector *S*. *enterica*, thus promoting proliferation and dispersal of bacteria within poultry associated environments [9, 10]. To a similar extent, previous studies showed that infestations of the Aster leafhopper (*Macrosteles quadrilineatus*) and the Green peach aphid (*Myzus persicae)* are associated with mechanical transmission of *S*. *enterica* to uncontaminated lettuce leaves [8]; interestingly, Aster leafhoppers were also found to ingest *S*. *enterica* and disperse the pathogen in honeydew [8]. In addition to assisting the movement of bacteria, phytophagous insect infestation on *S*. *enterica-*contaminated plants led to a prolonged persistence of the bacteria when compared to plants without insects [5, 11]. While the exact mechanisms causing prolonged persistence of the bacterium are unknown, unique insect feeding behaviors and associated damage to plants are suspected to provide a favorable environment for *S*. *enterica* persistence by providing a more direct route of entry to the apoplast of plant leaves, protected leaf interior, and/or to damaged cells leaking nutrients.

Western flower thrips, *Frankliniella occidentalis* Pergande (Thysanoptera: Thripidae) are considered a highly polyphagous insect species and employ a modified form of piercing-sucking feeding, whereby the stylets do not reach the vascular system of plants to withdraw vascular constituents [12]. Instead, adult, and immature *F*. *occidentalis* feed by initially puncturing surface mesophyll cells and then sucking out cellular contents. In this study, we tested the hypothesis that areas of leaves with macroscopic feeding damage caused by adult *F*. *occidentalis* support greater *S*. *enterica* populations than undamaged areas. Additionally, we investigated whether insect containment or open infestation of *F*. *occidentalis* would differentially affect the magnitude of leaf damage and *S*. *enterica* survival. We also postulated that the gender of *F*. *occidentalis* could influence the survivorship of *S*. *enterica*¸ similar to the results observed with transmission of *Tomato spotted wilt tospovirus* [TSWV; 13, 14], where adult male *F*. *occidentalis* possessed higher transmission efficiencies compared to females. The findings herein elucidate whether insect feeding behaviors, namely feeding damage, locally promotes the persistence of *S*. *enterica* populations.

## Materials and methods

### Bacterial strains and culture conditions

A kanamycin (Kan) resistant strain of *S*. *enterica* serovar Typhimurium 14028s used in this study was grown in lysogeny broth (LB; Difco, Dickinson and Company, Sparks, MD) at 37°C with shaking at 200 rpm.

### Insect rearing

Colonies of *F*. *occidentalis* were maintained on green bean pods (*Phaseolus vulgaris*), where the insects utilize the pods as both an ovipositional substrate and a food source. A starter colony of *F*. *occidentalis* was provided by Dr. Thomas L. German, Professor Emeritus, University of Wisconsin, Madison, from a long-term colony maintained in their laboratory. Weekly, green bean pods were sterilized in a 5% Clorox solution for 15 min and rinsed twice with sterile water to remove potential pesticide residue prior to placement in rearing containers. Populations of *F*. *occidentalis* were maintained in 0.4 L plastic containers (Dart Container Corporation, Mason, MI) and held under a constant temperature (27°C) and a 16:8 (Light:Dark) photoperiod. Each container included a sheet of filter paper to prevent moisture from accumulating. Voucher specimens of adult female and male *F*. *occidentalis*, obtained from the original colony, have been deposited in the Wisconsin Insect Research Collection, University of Wisconsin-Madison (http://labs.russell.wisc.edu/wirc/).

### Insect infestation and plant inoculation experiments

To investigate whether plant age together with *F*. *occidentalis* infestation density would alter *S*. *enterica* populations and electrolyte leakage, a no choice arena experiment was performed. *Solanum lycopersicum* (tomato, cv. Money Maker) seedlings were cultivated using Professional Growing Mix (Sunshine Redi-earth) in 6” pots held in a growth room maintained at a 16:8 (L:D) photoperiod and 24°*C* light and 19°*C* dark conditions for three and five weeks. In a 2X2 factorial design, replicate sets of adult *F*. *occidentalis* were transferred onto six separate three and five-week-old tomato plants, at densities of 5 (low density) or 20 (high density) individuals per plant. Each plant was contained in a 15.5 cm (ht) and 10 cm (diameter) Plexiglas tube, fashioned with thrips-proof screening (Green-tek Inc., Janesville, WI) at one end of the tube to prevent insects from escaping the experimental arena, and infested cages were held at 26°C temperature with a 16:8 (L:D) photoperiod. Seventy-two hours after the initial release of insects into cages, all adult *F*. *occidentalis* were removed, and the number of feeding lesion sites were visually assessed and counted. Subsequently, each plant was dip-inoculated in 450 ml of sterile water or a 10^8^ CFU/ml suspension of *S*. *enterica* (each beaker containing 75 μL of Sil-Wet, a surfactant aiding solution adhesion) for one minute. For bacterial inoculum preparation, lysogeny broth was inoculated with bacteria from -80°C freezer stocks and incubated, shaking overnight at 37°C. Bacterial cultures were normalized to an optical density at 600 nm of 0.2 in sterile water. Inoculum populations were verified by enumerating populations following serial dilution, plating on LB-Kan agar (50 μg/ml), and growth overnight at 37°C. Dip-inoculated plants were then placed in a clear, uncovered plastic bin at 27°C temperature under a 16:8 (L:D) photoperiod.

To assess *S*. *enterica* populations, plants were sub-sampled 72 hours after dip-inoculation to determine whether bacterial populations were localized to damaged sites on leaves. We chose to examine *S*. *enterica* population dynamics 72 hours post inoculation, as bacteria begin to diverge in population size within the presence or absence of insect infestation upon tomato leaves at this point [5]. Specifically, one 10 mm diameter leaf disc with macroscopic feeding damage (silvering), and one leaf disc absent of visible (macroscopic) feeding damage were extracted from the same leaf on dip-inoculated plants 72 hours following inoculation. These samples derived from the 2X2 factorial design were individually homogenized in 500 μL of sterile water using a cordless Dremel tool, and further diluted 1:10 in sterile water. Homogenates were immediately plated on LB-Kan agar, incubated overnight at 37°C and enumerated after 24 hours. Experiments were performed with three biological replicates.

To further characterize the magnitude of cellular or leaf damage associated with 72-hour thrips infestation, measurements of electrical conductivity were obtained from a comparable set of three, 10 mm-diameter leaf discs with macroscopic feeding damage, and three visibly undamaged leaf discs from the same leaf on each of three plants. Each group of three leaf discs were individually placed in single wells of a 12-well tissue culture plate containing 4 ml of sterile water. Plates were positioned on a rotating table at 50 rpm for approximately 30 min, acting as a wash step to prevent remnant soil particles from influencing conductivity measurements [15]. Subsequently, water from each well was removed and replaced with fresh, sterile water, and electrical conductance was immediately measured. Electrical conductance was measured by pipetting 1 ml of water from sample wells onto a ECTestr11+ MultiRange electrical conductance probe to assess the extent of conductive solute leakage, here used as a proxy for cellular damage. After the initial assessment of electrical conductance, sample plates were left covered under light banks at an ambient temperature for 6 hours, after which a second and final conductivity measurement was taken. Measures of electrical conductance were calculated by subtracting between the two time points (initial and second) and were used to evaluate the extent of electrolyte leakage over a six-hour period. Differences in measured conductance were used for data analysis for each plant age and infestation density treatment group.

An additional, free choice experiment was performed to assess if *F*. *occidentalis* would similarly influence electrical conductivity estimates and *S*. *enterica* populations compared with results observed in the no choice experiments. Briefly, adult populations (females and males) of *F*. *occidentalis* were released onto tomato seedlings for infestation periods of either three, four, or five weeks, resulting in plants infested with various life stages of the insects and obvious sites with feeding damage (silvering). This release of *F*. *occidentalis* onto plants allowed insects to actively move and thereby feed wherever they chose, hence the name ‘free choice experiment’. After the initial free-choice infestation period, 24 plants were randomly selected and removed from the experimental arena at one of the three time points post infestation and were dip inoculated into either a 450 ml aliquot of sterile water, or a 10^8^ CFU/ml suspension of *S*. *enterica* (each beaker containing 75 μl of Sil-Wet) for one minute. Bacterial cultures were prepared as described above. Replicate sets of 10 mm-diameter leaf discs were extracted 3 days after dip inoculation from separate leaves on each of 24 biological replicates per time point following infestation. Electrical conductance and *S*. *enterica* populations were assessed as described previously.

To determine whether insect gender influences *S*. *enterica* population dynamics, adult populations of male or female *F*. *occidentalis* were provided access to separate plants. Six, 2 cm diameter, thrips-proof, Plexiglas clip cages were attached to the underside of three, middle *S*. *lycopersicum* leaflets on opposing leaves. Three clip cages were infested with three individual male or female *F*. *occidentalis*, whereas the remaining three clip cages remained empty. Three days post infestation, insects and clip cages were removed, and each plant was subsequently dip inoculated into either a 450 ml aliquot of sterile water, or a 10^8^ CFU/ml suspension of *S*. *enterica* (each beaker containing *75* μl of Sil-Wet) for one minute. Bacterial cultures were prepared as described above. Replicate sets of 10 mm-diameter leaf discs were extracted 3 days after dip inoculation from separate leaves on each of the 4 individual plants following infestation by male or female thrips, and plants which remained absent of infestation. Electrical conductance and *S*. *enterica* populations were assessed as described previously. Experiments were performed with three biological replicates.

### Biotic vs. abiotic damage

To better understand the interaction between biotic (*F*. *occidentalis*) and abiotic cellular damage and resulting *S*. *enterica* populations, water pressure was used to inflict direct, physical (abiotic) damage to *S*. *lycopersicum* leaves, and served as a reference control. Three leaflets, from five-week-old plants randomly chosen for infestation, were faceted with three clip cages and infested with three adult *F*. *occidentalis*, whereas the remaining three clip cages on the opposite leaf remained empty on each plant. Additionally, six empty clip cages were applied to six leaflets between two opposing leaves for plants used as a control. At 72 hours after infestation, an inserted color cup was attached to a single action, siphon feed airbrush set (Paasche Airbrush Co., Kenosha, Wisconsin) and filled with sterile water, and a new set of five-week-old plants were randomly selected to receive short duration (5 sec) pulses of water at different pressures of 0.35, 1.41, or 3.78 kg cm^-2^ and directed to the underside of three middle leaflets. After inducing pressurized water damage, insects and clip cages were removed, and each plant was subsequently dip inoculated into either a 450 ml aliquot of sterile water, or a 10^8^ CFU/ml suspension of *S*. *enterica* (each beaker containing 75 μl of Sil-Wet) for one minute. Bacterial cultures were prepared as described above. Replicate sets of 10 mm-diameter leaf discs were extracted three days after dip inoculation from separate leaves on each of the 4 plants per treatment group following infestation. Electrical conductance and *S*. *enterica* populations were assessed as described previously. Experiments were performed with three biological replicates. To examine cell membrane viability of areas damaged by *F*. *occidentalis* or water pressure application, whole leaves were extracted 72 hours after imposed damage (as previously described) and immediately submerged in 10 mL of 0.25% Evans blue dye and incubated on a rotating table at 80 rpm for 20 minutes [16]. Following incubation, whole leaves were rinsed with sterile water to remove residual dye and observed with a Leica MZFL3 stereoscopic microscope.

### Statistical analysis

A one-way, analysis of variance (ANOVA) was used to assess if *S*. *enterica* populations or electrical conductance measurements varied between damaged and undamaged leaf discs derived from plants varying in age (3 or 5 weeks old), subjected to different *F*. *occidentalis* infestation densities (5 or 20 adult thrips), subjected to male or female infestation, and when damaged by *F*. *occidentalis* or water pressure. Student’s t-tests were performed to determine if feeding damage prompted a difference in electrical conductance of *S*. *enterica* populations when compared to undamaged plant tissues regardless of their treatment. To analyze the same response, student’s t-tests were also applied to the free-choice experiment. Outliers within each experiment were kept.

## Results

### *S*. *enterica* populations and electrolyte leakage are greater in *F*. *occidentalis* damaged sites in no-choice experiments

**Fig 1:**
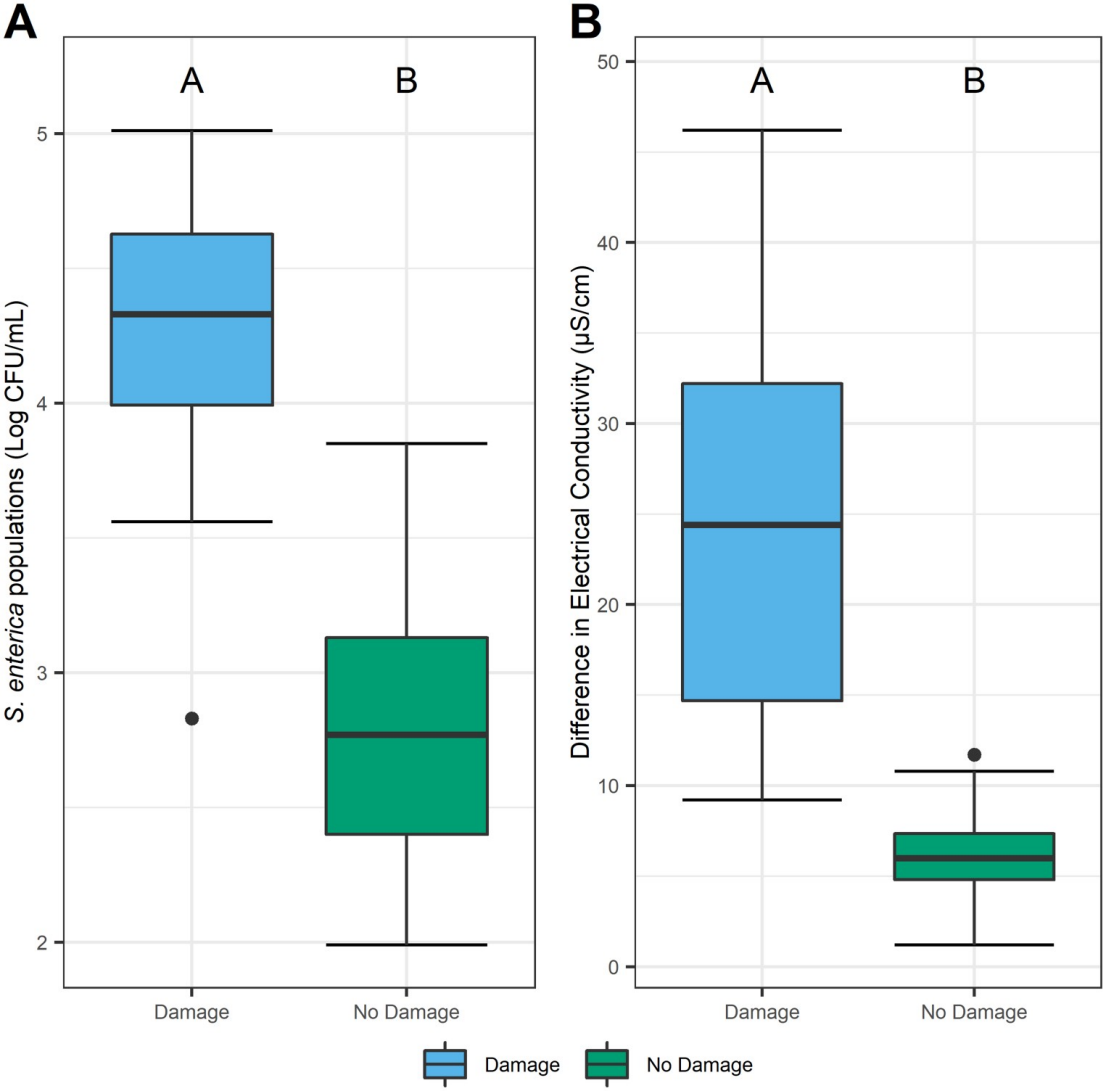
Impact of *F*. *occidentalis* damage on *S*. *enterica* populations and electrolyte leakage in a confined environment. In a no-choice experimental arena, tomato leaf areas with *F*. *occidentalis* damage (blue) had higher *S*. *enterica* populations (A) and greater differences in estimated electrolyte leakage (B) than undamaged sites (green). Means from each treatment group represent combined responses over plant age (3 or 5 weeks) and infestation density (5 or 20 insects per plant), as these main effects were non-significant. Measures of electrical conductance were calculated by subtracting the final from the initial measurement for damaged and undamaged leaf discs and were used to evaluate the extent of electrolyte leakage over a six-hour period. Boxplots with different letters indicate a significant difference (*P* <0.05), as determined by a student’s t-test. Singular dots represent outlier points.

To learn whether populations of *S*. *enterica* were directly influenced by localized macroscopic damage sites from insect feeding, bacterial population dynamics were assessed on damaged and macroscopically undamaged tomato leaves. In a controlled, no-choice environment, tomato plants from two age groups (3 or 5 weeks-old) were exposed to low (5 insects/cage) or high (20 insects/cage) densities of *F*. *occidentalis* to investigate if plant age and insect density influenced *S*. *enterica* populations. Within each treatment group, *S*. *enterica* populations were 1 log higher on macroscopically damaged leaf discs(*P* < 0.0001; Fig 1A). To ascertain whether cellular damage co-occurred with *S*. *enterica* populations, electrical conductance was measured. Electrolyte leakage doubled on leaf discs in association with feeding damage (*P* < 0.0001; Fig 1B).

**Fig 2:**
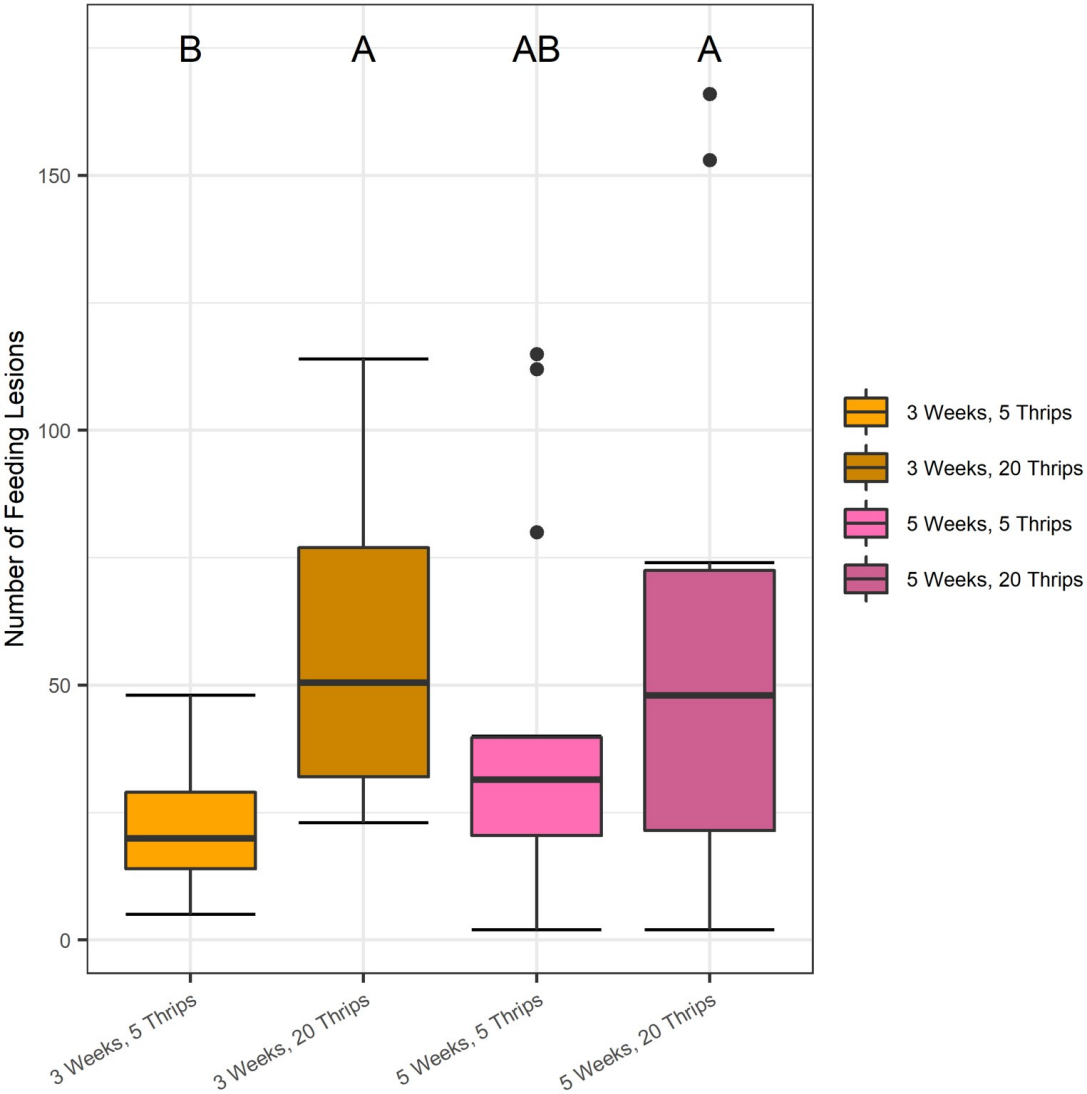
Quantity of *F*. *occidentalis* feeding lesions on tomato plants. In a no-choice experimental arena, younger (3 weeks-old) tomato plants with a greater, initial *F*. *occidentalis* infestation density (20 thrips/plant) exhibited significantly more individual feeding lesions than younger plants with lower, initial infestation densities (5 thrips/plant). Macroscopic feeding damage was visually assessed and counted after removing insects from each plant following a three-day infestation period. Data from each experimental replicate were combined and represented. Boxplots with different letters indicate a significant difference (*P* <0.05), as determined by a one-way ANOVA test. Singular dots represent an outlier point.

Next, we examined plant age and insect density. There was no significant differences when comparing treatment groups (variation in plant age and insect density) between macroscopically damaged leaves when assessing bacterial populations and electrolyte leakage (*P* > 0.05.; S1 Fig). Younger (3 weeks-old) tomato plants infested with higher, initial *F*. *occidentalis* populations densities (20 insects/cage) had twice as many macroscopic damaged areas when compared with plants exposed to lower infestation densities. (*P >* 0.05; Fig 2). However, older (5-week-old) tomato plants had similar numbers of feeding lesions regardless of initial *F*. *occidentalis* populations densities. Furthermore, the electrolyte leakage observed was similar to the results for the numbers of feeding lesions with more leakage on leaves exposed to more insects at 3 weeks but similar leakage on leaves at 5 weeks, independent of insect density (*P >* 0.05; S1 Fig).

**Fig 3:**
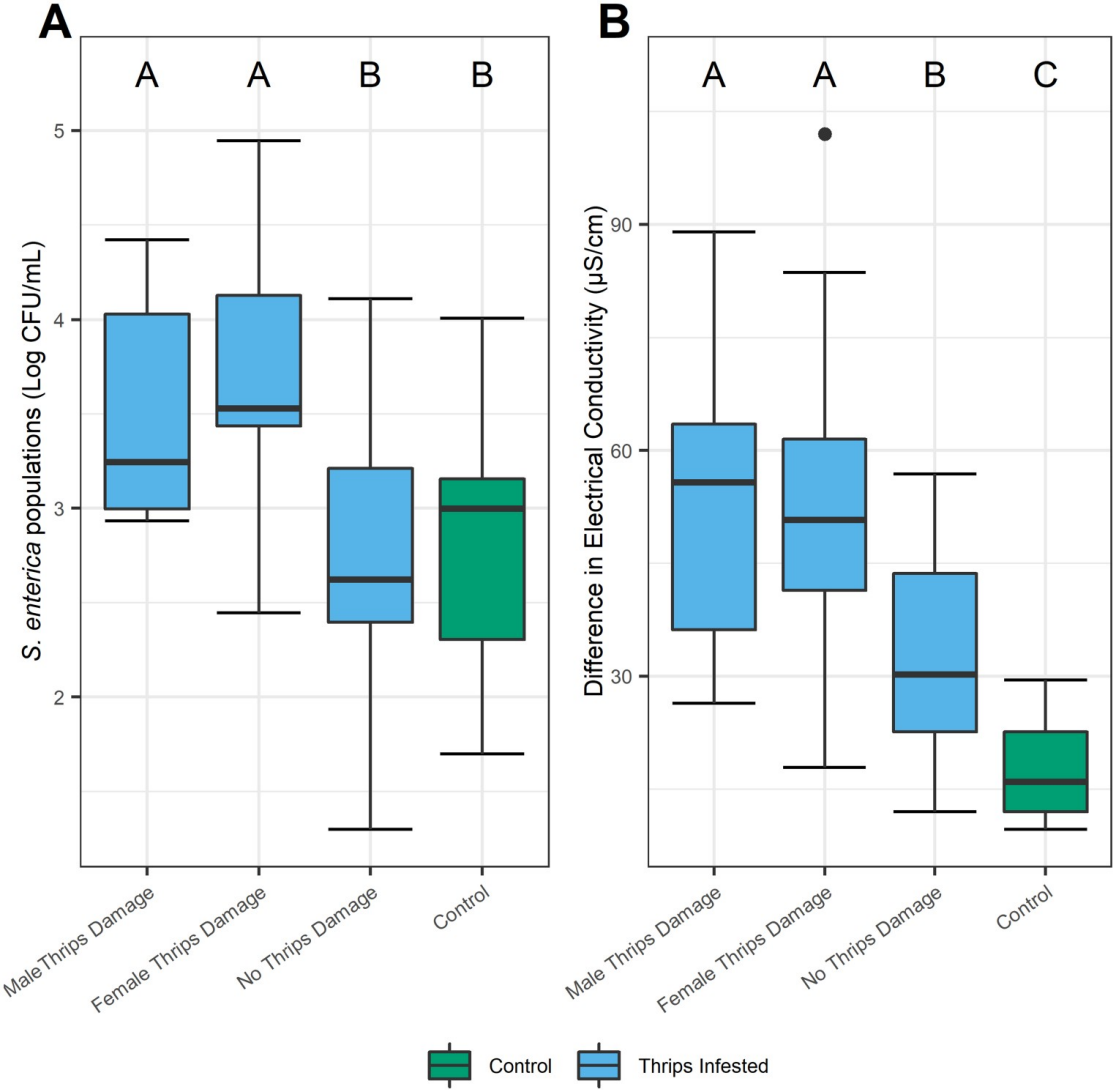
Male and female thrips damage enhances *S*. *enterica* populations and electrolyte leakage in a no-choice environment. Tomato leaf areas with *F*. *occidentalis* damage had significantly higher *S*. *enterica* populations (A) and significantly greater differences in electrolyte leakage (B) than undamaged sites. *S*. *enterica* population dynamics and electrolyte leakage were not significantly different between males and females. ‘No Thrips Damage’ represents undamaged leaf discs from *F*. *occidentalis* infested plants, whereas the ‘Control’ represents undamaged leaf discs from uninfested plants. Undamaged samples (No Thrips Damage) from plants previously infested by males or females were combined and means are represented. Measures of electrical conductance were calculated by subtracting the final from the initial measurement for damaged and undamaged leaf discs and were used to evaluate the extent of electrolyte leakage over a six-hour period. Boxplots with different letters indicate a significant difference (*P* <0.05), as determined by a one-way ANOVA test. Singular dots represent an outlier point.

We also examined insect gender. In an additional no-choice experiment, tomato leaves damaged by male or female *F*. *occidentalis* had significantly greater electrolyte leakage (*P* < 0.0001) and higher *S*. *enterica* populations (*P* < 0.0001), when compared with visibly undamaged tissues on infested plants, or plants entirely absent of insects (Fig 3A and 3B). *S*. *enterica* populations and electrolyte leakage were not significantly different between plants exposed to male or female insects (*P* > 0.9801, *P* > 0.9628; Fig 3A and 3B). Surprisingly, visibly undamaged leaf discs excised from infested plants had double the amount of electrolyte leakage than uninfested plants (*P* < 0.0208; Fig 3B).

**Fig 4:**
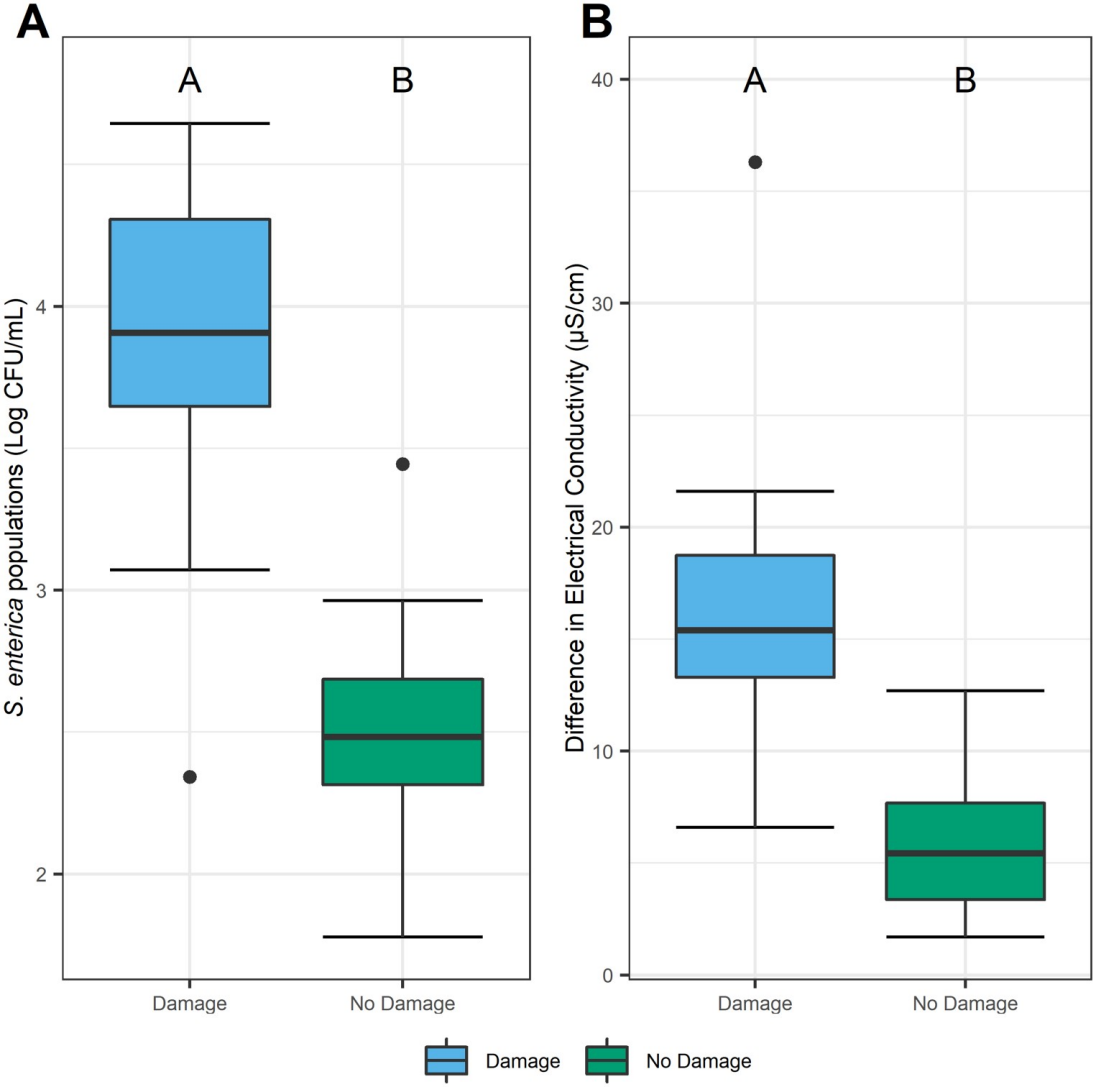
Thrips damage enhances *S*. *enterica* populations and electrolyte leakage in a free choice environment. In a free choice experimental arena, tomato leaf areas with *F*. *occidentalis* damage (blue) had higher *S*. *enterica* populations (A) and greater differences in estimated electrolyte leakage (B) than undamaged sites (green). Data from each infestation period of 3, 4 and 5 weeks were combined as *S*. *enterica* populations and electrolyte leakage estimates did not significantly vary over the experimental time periods. Measures of electrical conductance calculated by subtracting the final from the initial measurement for damaged and undamaged leaf discs were used to evaluate the extent of electrolyte leakage over a six-hour period. Boxplots with different letters indicate a significant difference (*P* <0.05), as determined by a student’s t-test. Singular dots represent an outlier point.

To model the natural environment of insect infestation and subsequent *S*. *enterica* plant contamination, we constructed a free choice experimental arena with various life stages of *F*. *occidentalis* infested tomato plants for experimental durations of 3, 4, or 5 weeks. *S*. *enterica* populations were a log greater and electrolyte leakage tripled on leaves exhibiting *F*. *occidentalis* feeding damage when compared to undamaged sites (*P* < 0.0001; Fig 4A and 4B, respectively).

### *S*. *enterica* populations are not exclusively dependent on cellular damage induced by *F*. *occidentalis*

**Fig 5:**
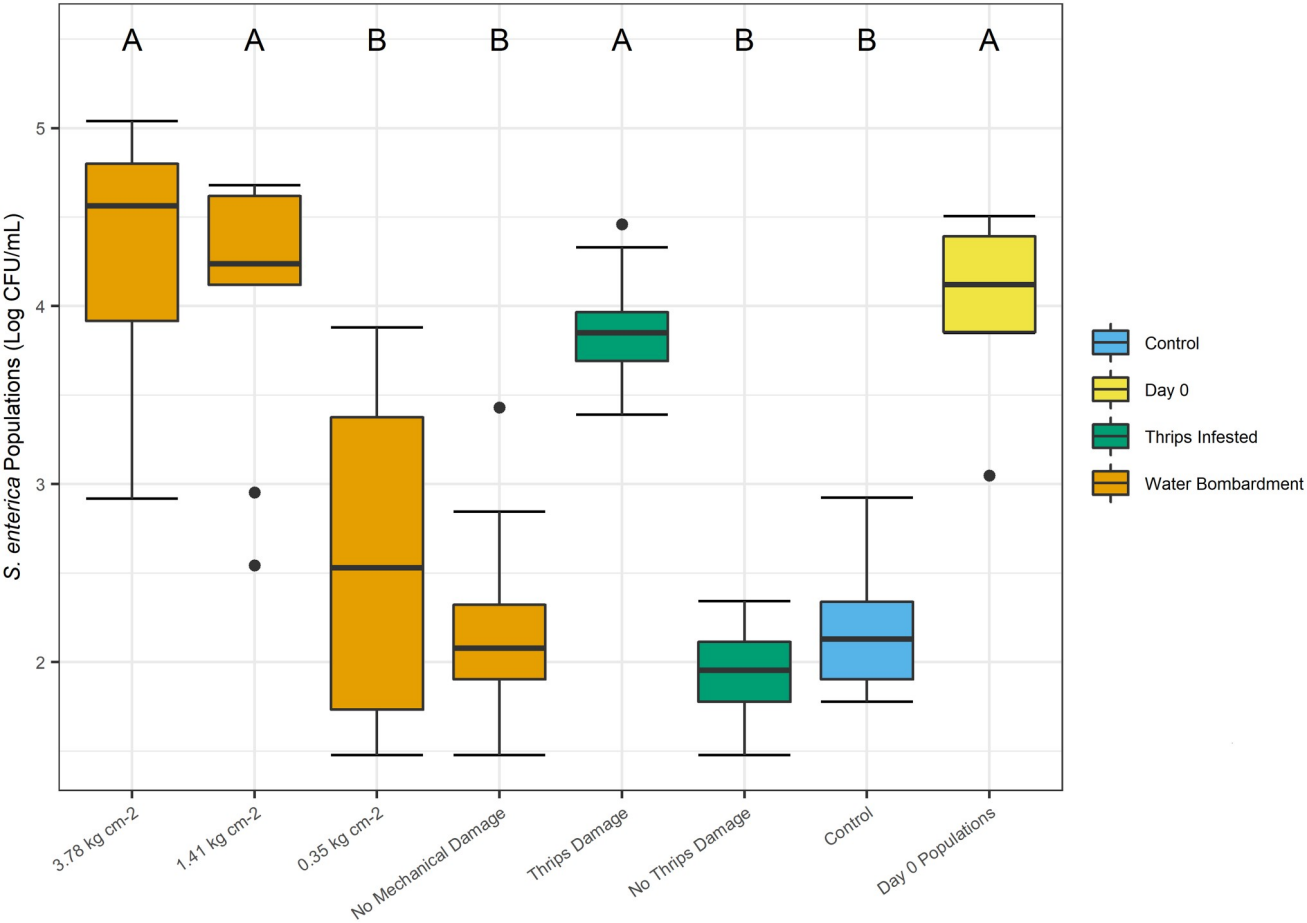
Impact of thrips damage and water bombardment on *S*. *enterica* populations. Tomato leaf tissues macroscopically damaged by adult *F*. *occidentalis*, or mechanically damaged by 1.41 or 3.78 kg cm^-2^ of water pressure, harbor significantly greater *S*. *enterica* populations than low pressure (0.35 kg cm^-2^) water treatments and undamaged leaves. Clip cages were fastened to each plant for three days containing three thrips or remained empty as a control. Three days after initial infestation, uninfested, or non-control, plants were subjected to mechanical damage induced by an airbrush paint atomizer, applying 0.35, 1.41 or 3.78 kg cm^-2^ of pressure for 5 seconds. After imposing the mechanical damage, each plant was dip-inoculated in a *S*. *enterica* solution for one minute and sampled for bacterial populations three days later. Three experimental replicates are represented, and different letters indicate significant differences between treatment groups (P < 0.05), as determined by a one-way ANOVA test.

**Fig 6:**
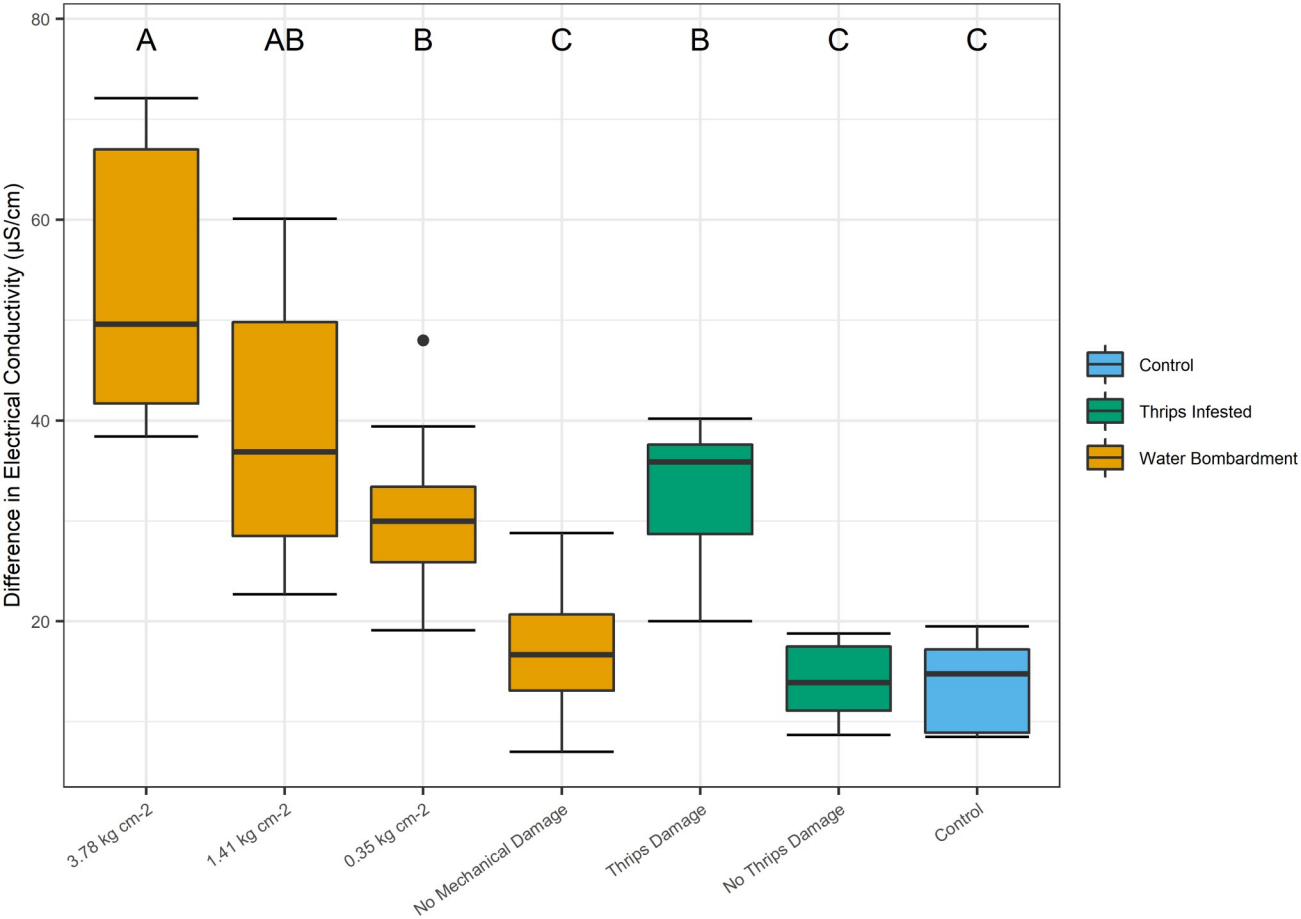
Impact of thrips damage and water bombardment on electrolyte leakage. Tomato leaf tissues macroscopically damaged by adult *F*. *occidentalis* feeding or mechanically damaged by 0.35, 1.41 or 3.78 kg cm^-2^ of water pressure for 5 seconds elicit significantly greater electrical conductance than undamaged leaves. Leaves damaged by 3.78 kg cm^-2^ of water pressure resulted in significantly greater cellular damage than leaves damaged by *F*. *occidentalis*. Clip cages were fastened to each plant for three days containing three thrips or remained empty. Three days post infestation, uninfested plants were subjected to mechanical damage induced by a paint atomizer, applying 0.35, 1.41 or 3.78 kg cm^-2^ of water pressure for 5 seconds. Measures of electrical conductance for damaged and undamaged leaf discs were used to evaluate the extent of electrolyte leakage over a six-hour period. Each treatment group represents twelve plants. Three experimental replicates are represented, and different letters indicate significant differences between treatment groups (P < 0.05), as determined by a one-way ANOVA test.

Cellular damage induced by insect feeding was hypothesized to be a factor that could enhance *S*. *enterica* population persistence. In this study, pressurized water inoculations were used as a reference control to represent abiotic, or physical damage in comparison to the biotic damage imposed by thrips feeding. First, we observed that *S*. *enterica* populations decreased over 2 logs within seventy-two hours following inoculation without insect or abiotic damage (*P* < 0.0001; Fig 5). Interestingly, leaves physically damaged by medium (1.41 kg cm^-2^) or higher (3.78 kg cm^-2^) water pressure, together with leaves possessing *F*. *occidentalis* feeding sites, exhibited *S*. *enterica* populations more than a log greater than leaves damaged by low water pressure (0.35 kg cm^-2^) or those without any form of visible damage (*P* < 0.0001; Fig 5). Leaves mechanically damaged by these varied pressures (0.35, 1.41, or 3.78 kg cm^-2^) resulted in significantly greater electrolyte leakage than undamaged leaves (*P* < 0.0106; Fig 6). Leaf tissue with *F*. *occidentalis* feeding sites resulted in significantly lower cellular damage than leaves damaged by high water pressure sprays (3.78 kg cm^-2^), indicating that the aggressive bombardment of water resulted in a greater amount of cellular damage in comparison to thrips feeding (Fig 6). Surprisingly, there was not an association between cellular damage and *S*. *enterica* populations; and of further interest *F*. *occidentalis* feeding caused significantly less cellular damage than high pressure sprays (3.78 kg cm^-2^), but resulted in equivalent bacterial populations.

## Discussion

Although *S*. *enterica* is traditionally studied in the context of animal hosts, the number of salmonellosis cases attributed to consumption of fresh produce warrants an assessment of environmental factors and how they promote bacterial food-borne outbreaks. On healthy plants, *S*. *enterica* lacks the necessary mechanisms to maintain phyllosphere populations on its own [17], most likely due to its inability to degrade plant cell wall components and thereby liberate nutrients from plant cells. Maintaining bacterial populations or growing on the leaf surface requires necessary enzymes to breakdown plant cells or alter the cell’s biochemistry. The prolific bacterial pathogen, *Pseudomonas syringae* for instance, produces biosurfactants that increase the rate of diffusion of water across plant cell cuticles subsequently prompting a release of foliar nutrients and making them successful, primary phyllosphere colonists [18]. Despite *S*. enterica*’s* inability to persist on its own, ubiquitous environmental stressors such as the presence of insects on plants, has previously been demonstrated to enhance *S*. *enterica* survival [5, 11], and thus increasing the risks for outbreaks of food borne illness from consumption of fresh produce. Although the fruits, of tomato plants are traditionally consumed and linked to food borne outbreaks, earlier investigations have previously isolated *S*. *enterica* from tomato fruit a month after initial *S*. *enterica* foliar inoculation. This affirms the relevance of assessing *S*. *enterica* populations upon tomato leaves in relation to food borne outbreak risks especially in the context of foliar feeding insects [19]. In the current study, we examined the tri-trophic interactions between *S*. *enterica*, *F*. *occidentalis*, and tomato plants, specifically investigating the effects of cellular damage induced by *F*. *occidentalis* on *S*. *enterica* populations, and further evaluating the influence of insect gender, plant age, and infestation density on bacterial populations.

In both choice and no choice experiments, we demonstrated that *S*. *enterica* population dynamics are influenced by insect feeding damage on tomato leaves (Figs 1 and 4). Prior research from the Barak and Groves laboratories determined that lettuce leaves previously damaged by *F*. *occidentalis* harbor greater *S*. *enterica* populations compared to undamaged areas. Here, we expand our understanding of *F*. *occidentalis* as a biological multiplier for *S*. *enterica* on a new host plant *L*. *solanaceae* and further demonstrate how the timing of bacterial contamination can follow insect damage. While feeding, *F*. *occidentalis* ingest plant cell contents [12], including the cytoplasm [20] and chloroplasts [21]. Epidermal and mesophilic leaf cells impacted by *F*. *occidentalis’* piercing-sucking mouthparts are generally emptied during ingestion [12]. It is plausible to assume, however, that the remaining, and newly exposed, plant cell constituents could consequently benefit epiphytic bacteria as a nutrient source. One effective way to quantify the proportion of newly exposed foliar contents is to analyze the extent of electrolyte leakage. In the event of damage or death, plant cells lose membrane integrity causing electrolytes to leak into the surrounding and exposed environment [22]. Thus far, plant pathogen attacks and hostile environmental conditions such as drought have been implicated as biological factors inducing electrolyte leakage [23, 24]. Electrolyte leakage caused by *Xanthomonas gardneri* infection resulted in *S*. *enterica* growth on tomato plants [17]. Previous studies, however, have not yet investigated if insect feeding behaviors elicit a similar and measurable response. In this study, we found that leaves with feeding damage exhibit significantly higher levels of cellular damage when compared with areas absent of feeding lesions (Figs 1, 3 and 4). Furthermore, an assay testing cell viability indicated that areas damaged by *F*. *occidentalis* exhibited a combination of destabilized and dead cells, demonstrating that damaged cells continue to leak constituents after feeding has ceased (S2 Fig). The plant-derived solutes released from damaged cells might provide sufficient metabolic requirements for *S*. *enterica* growth, as seen with the rich composition of exudates released by roots or germinating seeds [25]. To expand our understanding on this subject, future studies characterizing the composition of cellular and chemical leakage could further reveal the direct effects of *F*. *occidentalis* feeding on *S*. *enterica* populations.

Thrips feeding is distinct between males and females. Female *F*. *occidentalis* are defined as penetrative feeders, causing extensive scarring to epidermal and mesophyll cells in large concentrated areas, emptying out the entirety of cellular contents [26]. Males, on the other hand, are considered shallow feeders, lightly puncturing numerous epidermal and mesophyll cells, ingesting relatively small amounts of cellular contents and producing microscopic (invisible) scarring [14]. The gender of thrips has been shown to influence the transmission of viral pathogens, including *Tomato spotted wilt tospovirus* [TSWV; 13, 27]. Comparatively, females feed by devastating plant cells, which limit viral infection and subsequently replication. Although the influence of insect gender on virus transmission has been broadly investigated [28–30], no study has considered *F*. *occidentalis* gender when investigating human enteric bacterial pathogens on plants. In the case of *S*. *enterica*, we found that the gender of the insect had no significant influence on bacterial population dynamics (Fig 3), leading us to question whether cellular damage alone is the mechanism promoting *S*. *enterica* survival on thrips infested plants.

Plant and insect interactions have co-evolved strategies to best minimize damage between one another. Plants, for one, have evolved resistance and tolerance strategies against herbivores, actively mitigating the extent of damage (through biochemical or morphological means) or minimizing the impact on plant fitness respectively [31–33]. Each of these innate defense mechanisms are found in plants but are inversely proportional between juvenile (vegetative) and reproductive developmental stages, and thus, pre-flowering plants could be considered the intermediate in terms of confronting herbivores with plant resistance or tolerance strategies [34, 35]. In our study, we found that five-week-old plants had similar lesion numbers regardless of densities of insects. This observation indicates that *F*. *occidentalis* feeding behavior may change as pre-reproductive plants age since the extent of damage as measured by conductivity followed a similar pattern as numbers of lesions. Fewer insects could cause extensive damage as pre-reproductive plants age altering the phyllosphere to a more inhabitable environment for *S*. *enterica* as the plant begins production of the raw fruit commonly implicated in salmonellosis outbreaks.

**Fig 7:**
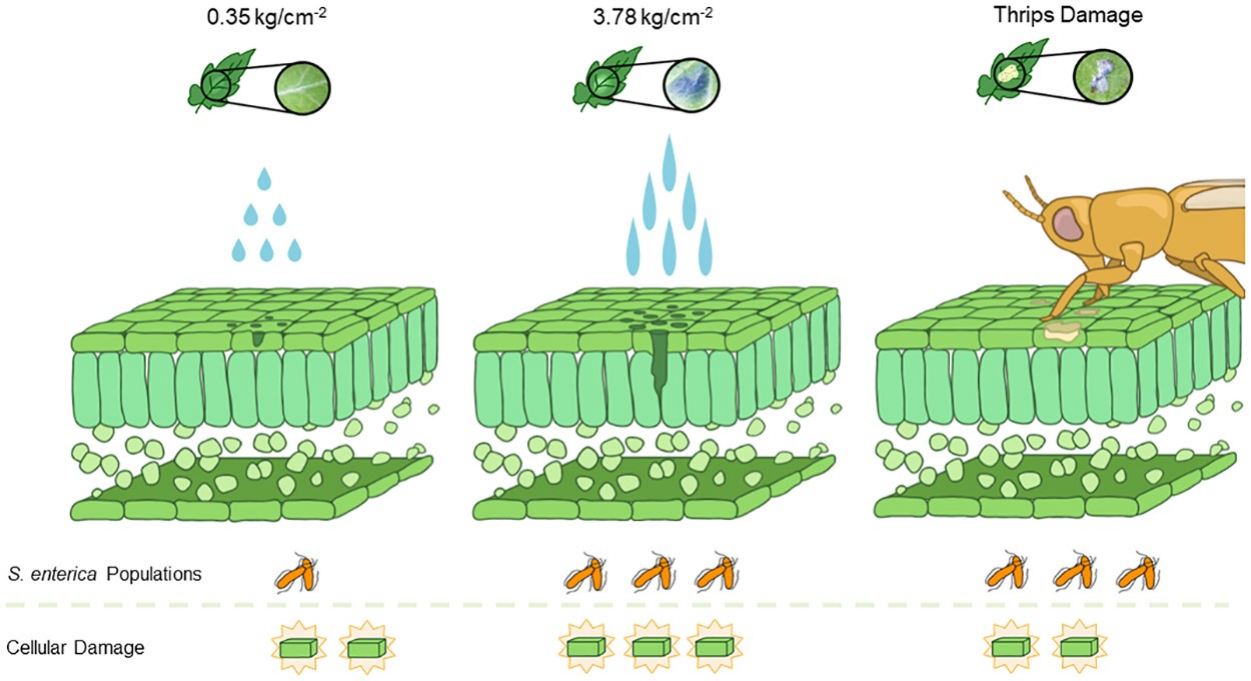
*Salmonella enterica* survival is not strictly correlated with the extent of cellular damage. The delivery of high-pressure water alone (middle) resulted in significantly greater amounts of cellular damage, when compared with areas possessing direct damage as a result of *F*. *occidentalis* feeding (right). Leaves damaged with lower water pressure (left) displayed significantly lower *S*. *enterica* populations in comparison to the two aforementioned groups. Regardless of the degree of cellular damage, *S*. *enterica* populations were similar between plants physically damaged by the highest water pressures and those compromised by *F*. *occidentalis* feeding, indicating that cellular damage is only one of the potential biological mechanisms which may enhance *S*. *enterica* populations in the plant phyllosphere.

We hypothesized that areas with greater damage, and thus a higher exposure to plant nutrients, would result in a higher overall *S*. *enterica* population. To better understand this interaction, we imposed varying levels of abiotic physical damage to emulate thrips feeding damage in isolation of other biotic interactions. Results from these experiments indicated that although an aggressive bombardment of water resulted in significantly greater cellular damage beyond that which we observed from *F*. *occidentalis* (Fig 6), *S*. *enterica* populations were similar across these levels (Fig 5). The data from this experiment suggests that *S*. *enterica* survival is not strictly correlated with the extent of cellular damage, as described and illustrated in our hypothetical model (Fig 7). Rather, there are likely other biological factors directly, or indirectly influenced by *F*. *occidentalis* feeding which may enhance epiphytic *S*. *enterica* population persistence. One possible explanation may be an upregulation of an immune response to insect feeding damage. Recent investigations have shown that the co-occurrence of phytophagous insects on *S*. *enterica* inoculated plants results in an active, up-regulation of both jasmonic and salicylic acid defense pathways, benefiting *S*. *enterica* epiphytic populations [5]. Similar to investigations with Auchenorrhyncha leafhoppers [36], thrips have been shown to elicit an upregulation of jasmonic acid defense pathways in response to feeding and ingestion [37, 38], and thus, may benefit *S*. *enterica* populations in this indirect way.

In the context of agricultural ecosystems, we identified several important relationships between *F*. *occidentalis*, *S*. *enterica*, and the tomato phyllosphere. Our study indicated that growers may face a greater likelihood, and possibly a prolonged period of vulnerability to produce contamination with foliar feeding damage induced by thrips. More so, greater feeding damage likely indicates a greater proliferation, or protracted interval of risk of *S*. *enterica* suggesting appropriate pest management actions may be warranted where the risk of *S*. *enterica* and *F*. *occidentalis* co-occurrence is greatest, regardless of the sex ratios observed in field populations.

## Supporting information

S1 Fig Thrips damage enhances *S*. *enterica* populations and electrolyte leakage in a no-choice environment. In a no-choice experimental arena, damaged leaf tissue exhibited higher *S*. *enterica* populations (top) and greater electrolyte leakage (bottom), regardless of plant age or initial *F*. *occidentalis* infestation density. Damaged and undamaged leaf discs were extracted from each three or five-week-old plant with high (20 thrips/cage) or low (5 thrips/cage) infestation densities. Measures of electrical conductance for damaged and undamaged leaf discs were used to evaluate the extent of electrolyte leakage over a six-hour period. Boxplots with different letters within each treatment group indicate a significant difference (*P* < 0.05), as determined by a student’s t-test. Singular dots represent outlier points.
(TIF)

S2 Fig Evans blue staining of damaged tomato leaflets. Five-week-old tomato leaflets were subjected to *F*. *occidentalis* feeding for 72 hours, or an application of low (0.35 kg cm-2), medium (1.41 kg cm-2), or high (3.78 kg cm-2) water bombardment for five seconds. The blue dot on the drawn leaflet was the location where water pressure or contained thrips damage was applied. Whole leaves were extracted after imposed damage, and immediately dyed to visualize cell membrane viability.
(TIF)
