# Supplementary figures and images for: Frankliniella occidentalis facilitate Salmonella enterica survival in the phyllosphere
Source: PLoS One. 2021 Feb 19;16(2):e0247325. doi: 10.1371/journal.pone.0247325 (PMC7895381; doi:10.1371/journal.pone.0247325)

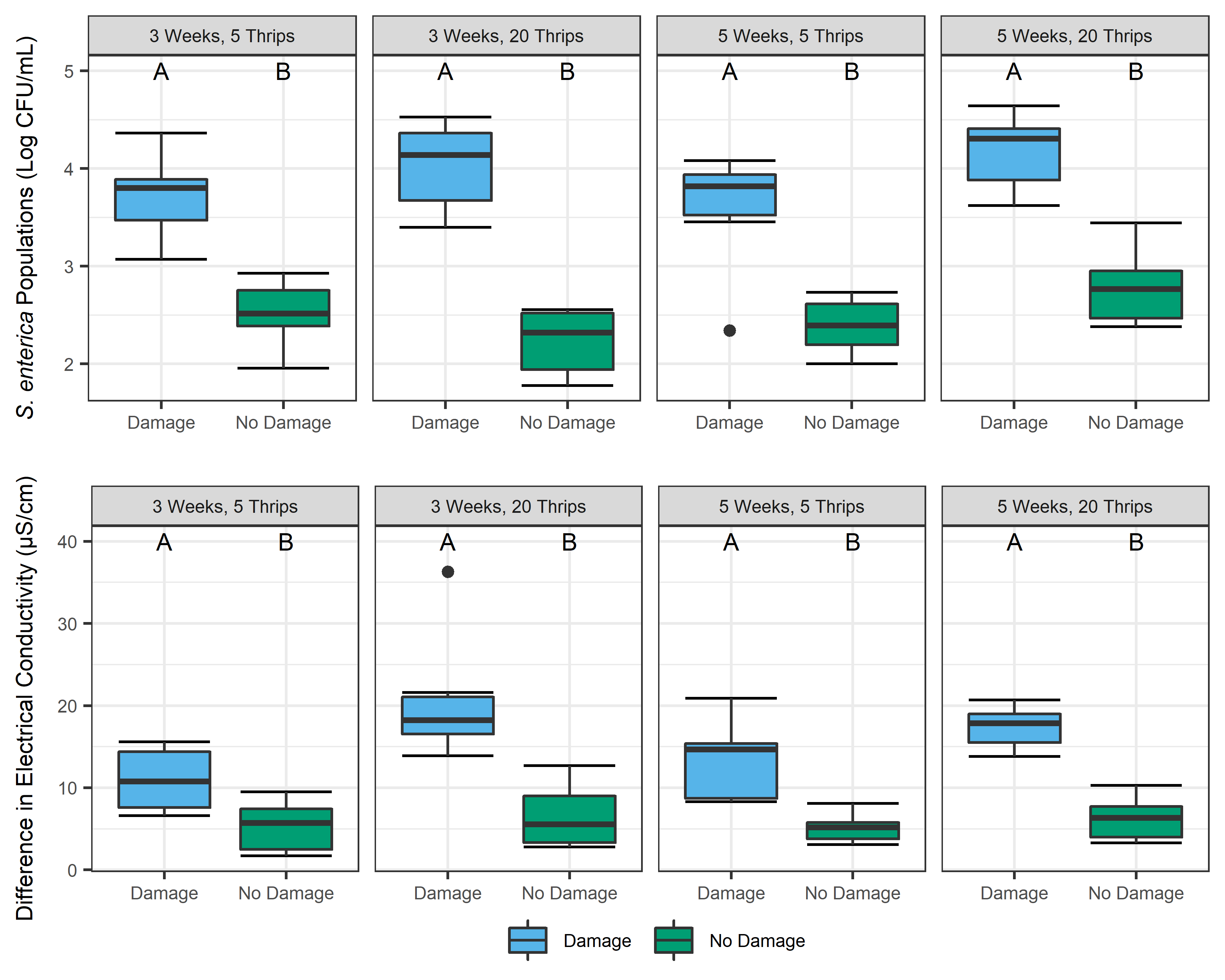

Supplement: S1 Fig — In a no-choice experimental arena, damaged leaf tissue exhibited higher S. enterica populations (top) and greater electrolyte leakage (bottom), regardless of plant age or initial F. occidentalis infestation density. Damaged and undamaged leaf discs were extracted from each three or five-week-old plant with high (20 thrips/cage) or low (5 thrips/cage) infestation densities. Measures of electrical conductance for damaged and undamaged leaf discs were used to evaluate the extent of electrolyte leakage over a six-hour period. Boxplots with different letters within each treatment group indicate a significant difference (P < 0.05), as determined by a student’s t-test. Singular dots represent outlier points. (TIF) [file pone.0247325.s001.tif]

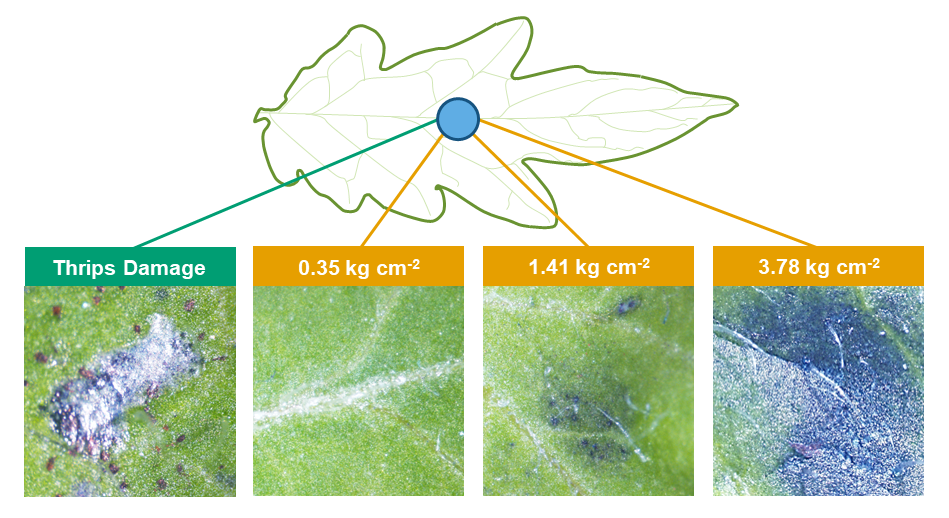

Supplement: S2 Fig — Five-week-old tomato leaflets were subjected to F. occidentalis feeding for 72 hours, or an application of low (0.35 kg cm-2), medium (1.41 kg cm-2), or high (3.78 kg cm-2) water bombardment for five seconds. The blue dot on the drawn leaflet was the location where water pressure or contained thrips damage was applied. Whole leaves were extracted after imposed damage, and immediately dyed to visualize cell membrane viability. (TIF) [file pone.0247325.s002.tif]
